# Mixed Co-Mn oxide coatings synthetized by DLI-MOCVD for SOC interconnect a parametric study for composition and homogeneity control

**DOI:** 10.1038/s41598-025-23783-5

**Published:** 2025-11-14

**Authors:** R. Chanson, F. Miserque, F. Schuster, F. Rouillard

**Affiliations:** 1 Service de recherche en Corrosion et Comportement des Matériaux (S2CM), Université Paris-Saclay, CEA, Gif-sur-Yvette, 91191 France; 2 Cross-Cutting program on advanced materials and manufacturing, Université Paris-Saclay, CEA, Gif-sur-Yvette, 91191 France

**Keywords:** Coating, Oxidation, MOCVD, Cobalt, Manganese, Spinel, Solid oxide electrolyzer cell, Green hydrogen, Chemistry, Energy science and technology, Engineering, Materials science

## Abstract

The present study investigates the synthesis of mixed cobalt manganese oxide coating for Solid Oxide Cell (SOC) interconnect application using DLI-MOCVD (Direct Liquid Injection-Metal Oxide Chemical Vapor Deposition). The possibility to control the coating thickness and composition by injecting a mixed solution of Co(acac)_3_ and Mn(acac)_3_ precursors is demonstrated. Various oxide compositions from Mn_x_Co_1-x_O to Mn_x_Co_3-x_O_4_ are synthesized. An acceptable homogeneity in terms of thickness and composition is possible over 25 cm along a tubular reactor which makes this process suitable for industrial application.

**Supplementary Information:**

The online version contains supplementary material available at 10.1038/s41598-025-23783-5.

## Introduction

High temperature Solid Oxide Cell (SOC) is a proposed technology to produce hydrogen with electricity and Solid Oxide Fuel Cell (SOFC) to produce electricity from hydrogen^1^. These systems are usually working between 700 and 800 °C. In this high temperature range, the volatilization of chromium oxide from stainless steel interconnect is evidenced to decrease the overall performance of Solid Oxide Cell^2^. Mn_x_Co_3−x_O_4_ spinel coating is identified as a good candidate to limit Cr diffusion and volatilization^3^. Moreover, it is evidenced that the electronic conductivity of Mn_x_Co_3−x_O_4_ depends on its Mn composition^4–7^: adding Mn in cobalt spinel increases its electrical conductivity with a maximum reached for x around 1. However, a high concentration of Mn in Co spinel favors Cr diffusion^8,9^ which is detrimental for its protection against Cr volatilization. In consequence, a compromise for the Mn/Co ratio in the coating must be found to reach a good electronic conductivity coupled with the ability of being a sufficient barrier to Cr diffusion^8^. Previous study shows up the capability to deposit cobalt and manganese oxide using spray pyrolysis and DLI-MOCVD (Direct Liquide Injection – Metal Organic Chemical Vapor Deposition)^10–13^. The capability to deposit {Co, Mn} mixed oxide coating with the injection of Co(acac)_2_ and Mn(thd)_3_ precursor in a pulsed spray evaporation system was also demonstrated in^14^. These coatings are synthetized in a cold wall reactor but the capability to control the coating composition directly with the solution composition in a system at the industrial scale has never been demonstrated in our knowledge. In this study, the capability to control the coating composition with the composition of the injected solution is demonstrated using a hot wall DLI-MOCVD reactor. The influence of the main process parameters such as temperature, pressure, O_2_ and precursor proportion in the gas phase on the coating composition, thickness and homogeneity along the 25 cm long reactor is studied and discussed.

## Methods

**Fig. 1:**
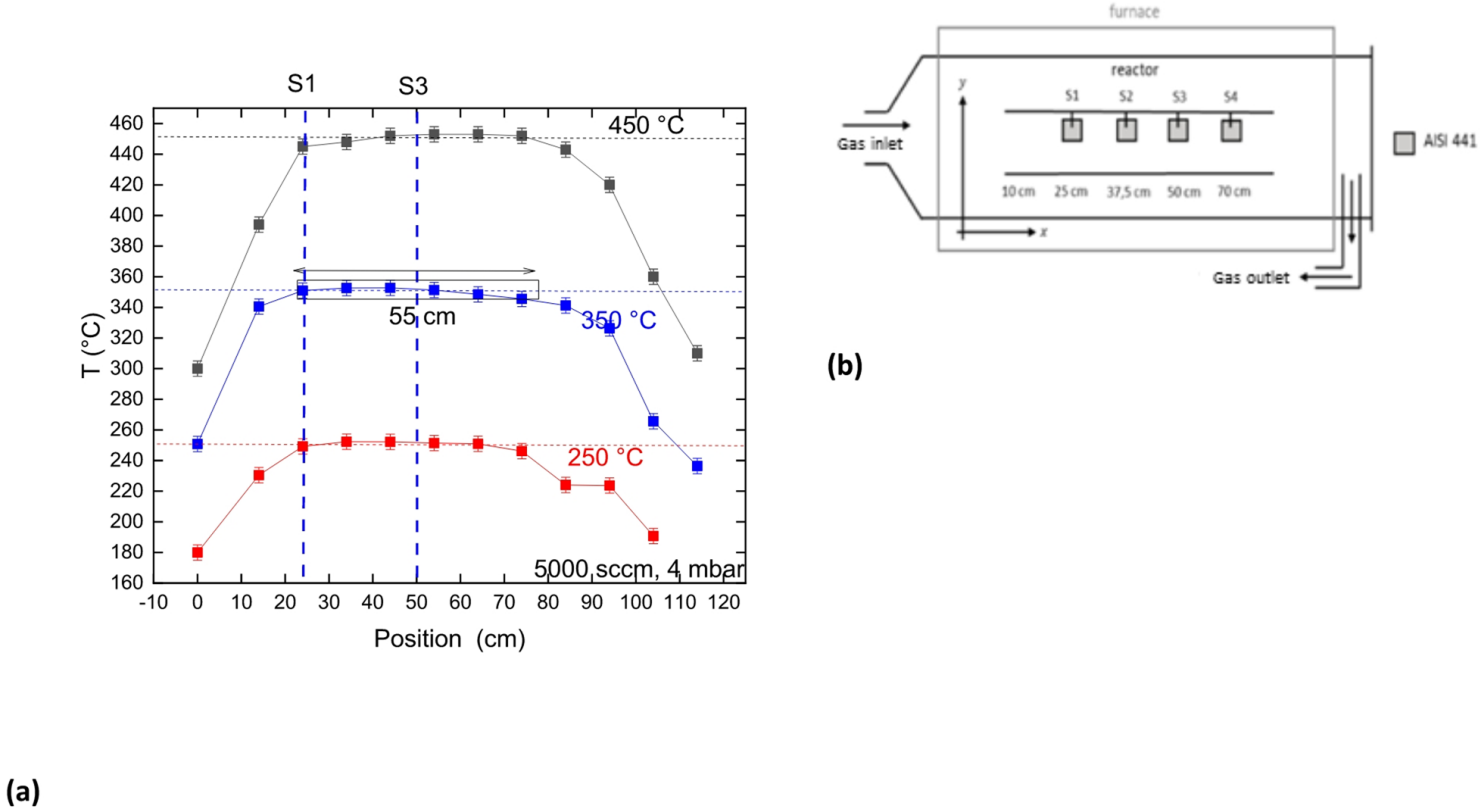
Temperature profile in the reactor for a setting temperature of 250 °C, 350 °C and 450 °C with 5500 sccm of N_2_ **(a)**. Scheme of the position of the samples in the reactor **(b)**.

Mn/Co oxide coatings are produced by DLI-MOCVD. In this coating process, a liquid solution containing Co and Mn organometallic precursors (in this case Co(acac)_3_ and Mn(acac)_3_ with “acac” for acetylacetonate) are diluted in a solvent (p-xylene) and injected in a vaporization box (VapBox from Kemstream^®^) with N_2_ as a gas vector. The gas phase formed in the Vapbox is then injected into a tubular reactor that is 1100 cm long and 15 cm in diameter heated by a three areas external furnace. The isotherm zone is defined to be the zone where the temperature keeps a constant value $\:\pm\:$ 5 °C. It is measured between 25 and 75 cm from the gas inlet (see Fig. 1a). O_2_ gas flow is added in the gas stream in order to react with Co(acac)_3_ and Mn(acac)_3_ to form oxide coatings on the sample surface. 200 μm thick AISI 441 stainless steel samples (30 $\:\times\:$ 30 mm^2^) are coated during 60 min. This stainless-steel grade contains about 18 wt. %Cr, ≤ 1 wt% Mn, ≤ 0.10–0.60 wt% Ti, ≤ 1 wt% Si, ≤ 0.03 wt% C, ≤ 0.015 wt% S, ≤ 0.04 wt% P, and ≤ 1 wt% Nb. Every batch is composed of 4 samples positioned in the center of the reactor radial axis at 25, 37.5, 50 and 80 cm from the gas inlet and are named respectively S1 (25 cm) to S4 (80 cm). The samples S1 and S3 are more deeply analyzed because the area between S1 and S3 is 25 cm long and are, therefore, good indicators of spatial coating homogeneity. The process parameters used for each batch are summarized in Table 1. The total gas flow is always 5500 sccm for all experiments. The coating thickness deposited on every sample is estimated by weight measurement assuming a coating density of 5.78$\:\:\pm\:\:$0.56 g.cm^−3^. The value of 5.78 is the average value of the density evaluated by XRD. The error bar is defined as the difference between the average value and the extremums of the density distribution. The nature, morphology and composition of the coatings are determined by grazing X-Ray Diffraction (XRD) at 4° incidence with a Brucker advance D8. SEM cross section has been performed with a Zeiss Gemini 2. The Glow Discharge-Optical Emission Spectroscopy (GD-OES) has been performed using a HORIBA GD-profiler 2 spectrometer. The discharge lamp works with an Argon partial pressure of about 850 Pa. The input power was adjusted to 60 W. The Cu anode diameter is 4 mm.

**Table 1:** Experimental conditions for the coated sample batchs.

Batch name	Setting temperature (°C)	*P* (mbar)	% O_2_	% prec (gas phase)	$\:\frac{\left[\varvec{C}\varvec{o}{\left(\varvec{a}\varvec{c}\varvec{a}\varvec{c}\right)}_{3}\right]}{\left[\varvec{M}\varvec{n}{\left(\varvec{a}\varvec{c}\varvec{a}\varvec{c}\right)}_{3}\right]}$ solution
B1	350	10	30	0.02	1
B2	400	10	30	0.02	1
B3	450	10	30	0.02	1
B4	450	10	50	0.08	1
B5	450	10	50	0.04	1
B6	450	10	50	0.02	1
B7	400	10	30	0.03	2
B8	400	10	30	0.03	0.5
B9	350	4	30	0.04	1
B10	350	10	30	0.04	1
B11	350	25	30	0.04	1
B12	350	42	30	0.04	1
B13	450	10	10	0.02	1

## Results

### Influence of temperature on the composition and thickness of Co-Mn oxide coatings

**Fig. 2:**
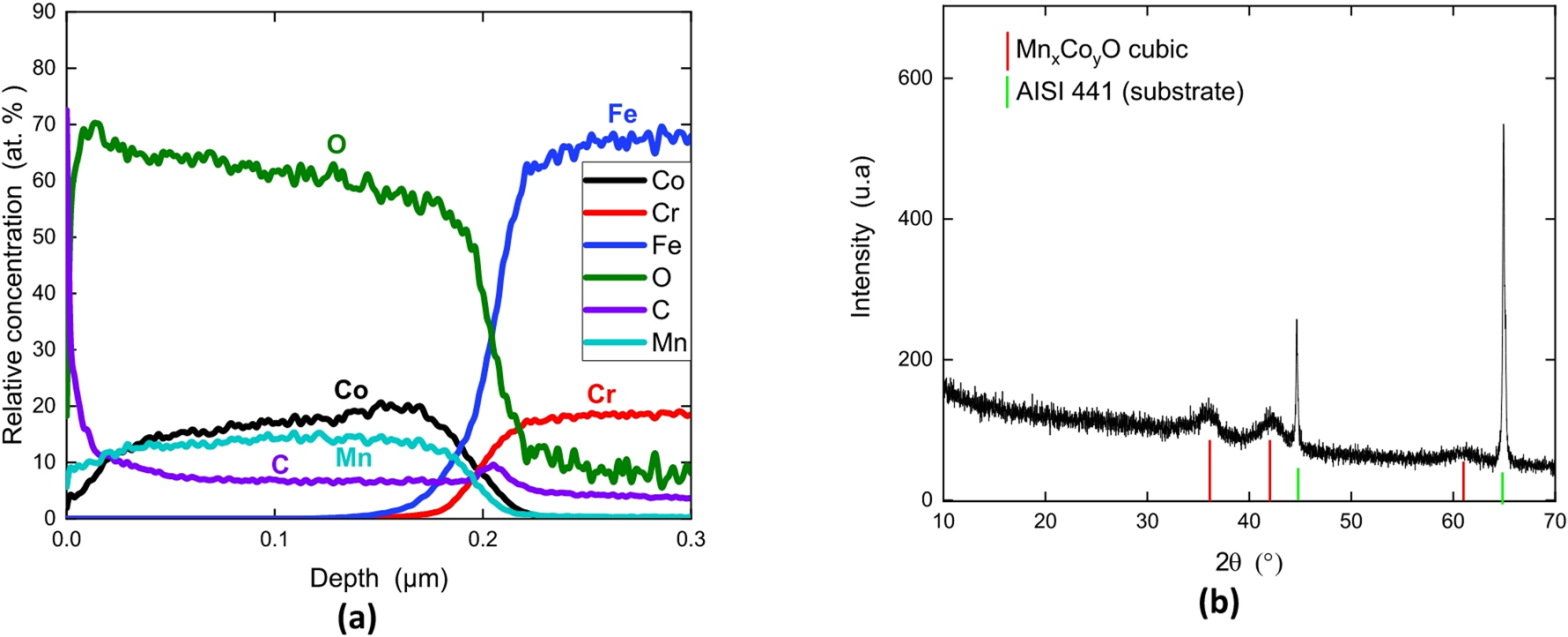
GD-OES quantitative depth profile **(a)** and XRD pattern **(b)** of the coating formed on sample S1 for batch B1 at 350 °C.

Figure 2 presents the GD-OES and the XRD pattern of sample S1 from batch B1 made at 350 °C. The coating is composed of Mn_x_Co_1−x_O oxide according to the UPDF2 database (Fig. 2b). The wide XRD peak suggest that the coating may be mostly amorphous. Another explanation could be that the coating is composed of nano-crystals or has lattice parameter dispersion owing to local composition evolution and/or a stress gradient despite the GD-OES shows a global composition homogeneity. The Mn/Co atomic ratio in the coating is different than the one in the solution. The coating contains a large amount of C (about 8 at%) inside (Fig. 2a).

**Fig. 3:**
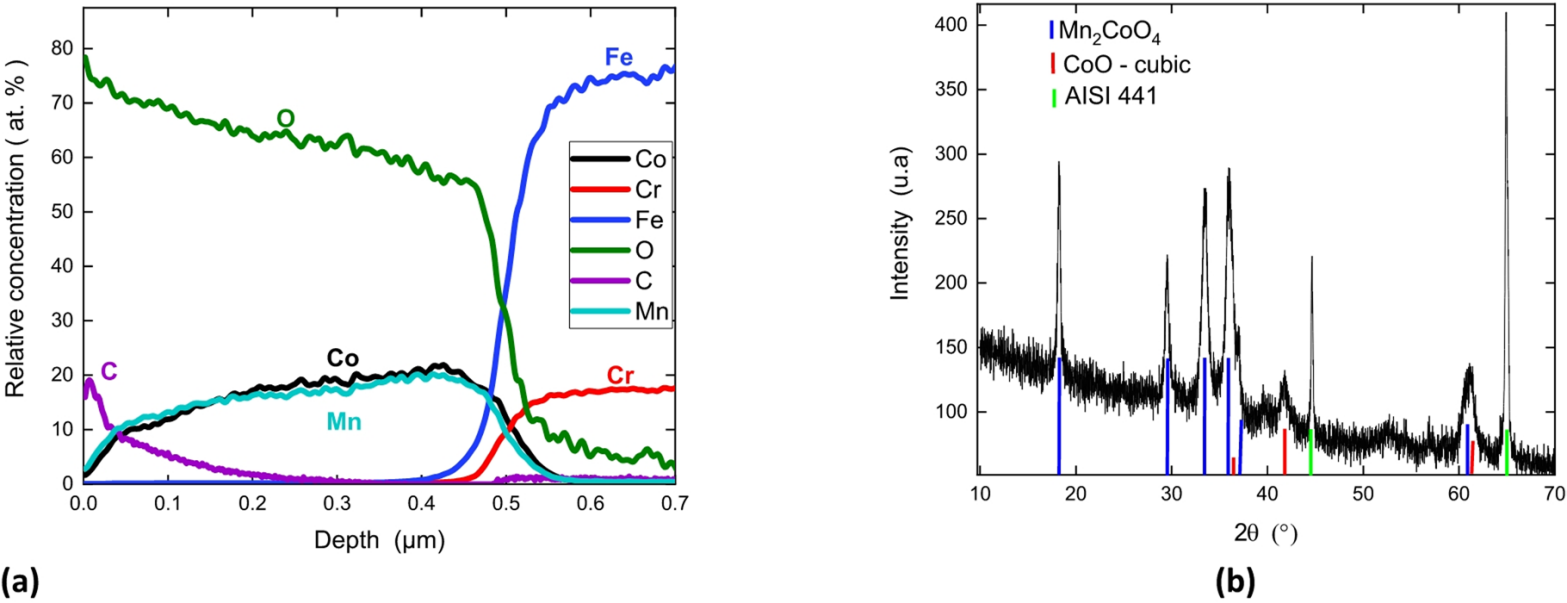
GD-OES quantitative depth profile **(a)** and XRD pattern **(b)** of the coating formed on sample S1 for batch B2 at 400 °C.

Figure 3 presents the GD-OES and the XRD pattern of sample S1 from batch B2 occurring at 400 °C. For this sample, Mn_2_CoO_4_ spinel phase with CoO cubic phase are identified revealing a higher degree of oxidation at higher temperature. The Co/Mn atomic ratio in the coating is equal to the one injected in the gas phase. The carbon concentration in the coating is lower than 3 at% (Fig. 2).

The GD-OES and the XRD pattern of the coating formed at 450 °C (batch B3) are very similar to the one formed at 400 °C and, for this reason, are not presented here.

**Fig. 4:**
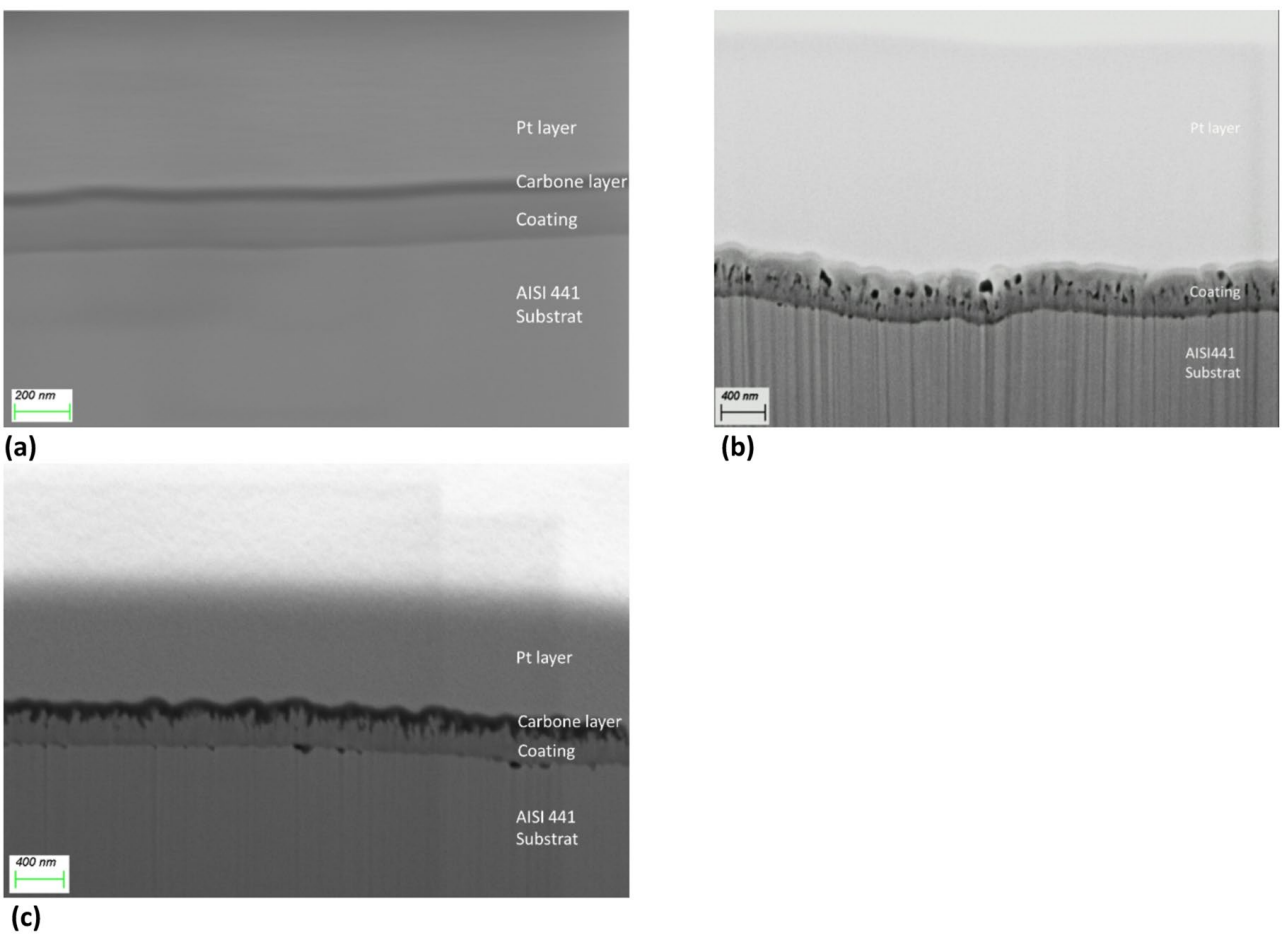
SEM cross-sections of S1 sample after Focused Ion Beam preparation from **(a)** batch B1 at 350 °C, **(b)** batch B2 at 400 °C **(c)** batch B3 at 450 °C.

Figure 4 presents the SEM cross section of the S1 sample **(a)** after batch B1 at 350 °C, **(b)** batch B2 at 400 °C and **(c)** batch B3 at 450 °C. At 350 °C, the coating appears smooth while at 400 and 450 °C, some roughness appears at the top of the structure. Moreover, a few pores are visible in the coating formed at 400 and 450 °C. The coating thickness measured by SEM cross section is 200 nm at 350 °C, 440 nm at 400 °C and 310 nm at 450 °C.

**Fig. 5:**
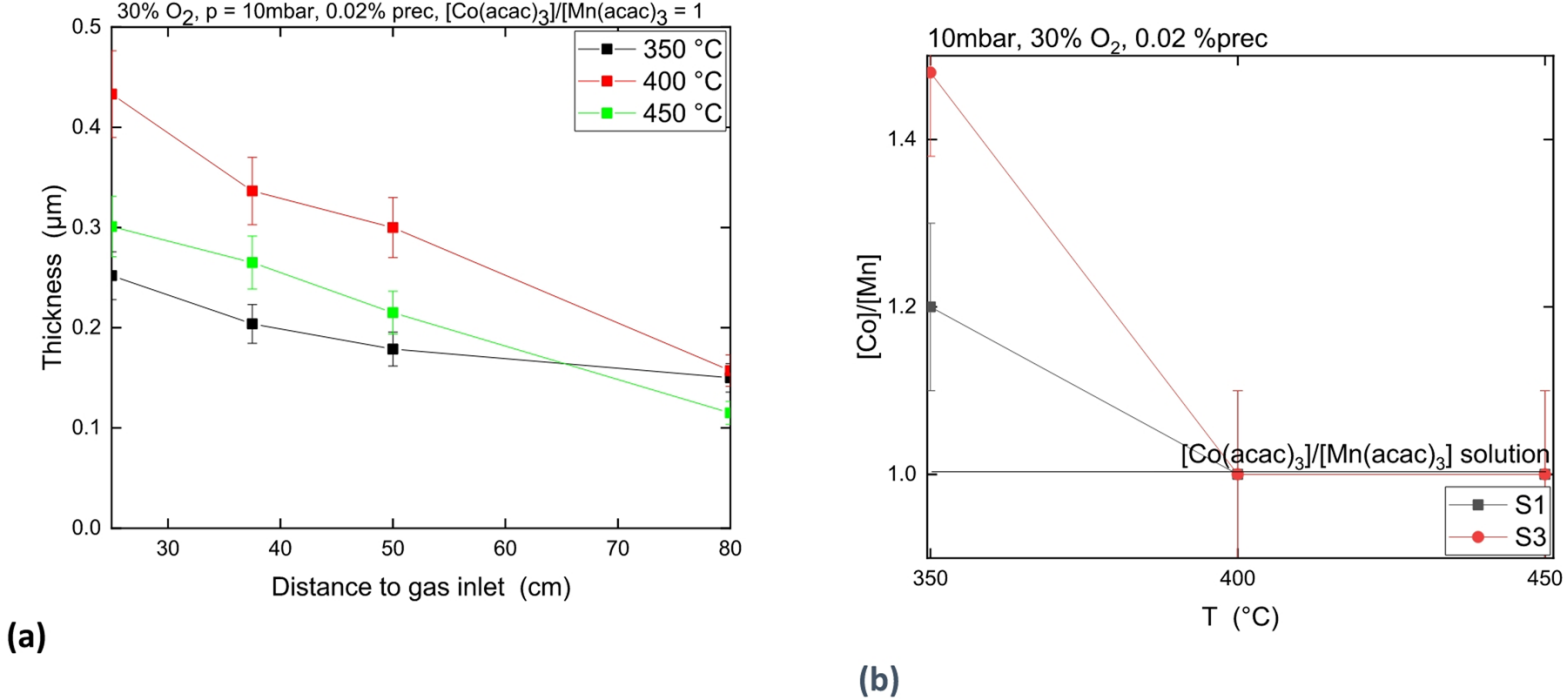
**(a)** Coating thickness as a function of the distance to gas inlet at various temperatures. **(b)** $\:\frac{\left[Co\right]}{\left[Mn\right]}$ atomic ratio measured in the coating by GD-OES as function of the temperature for S1 and S3 sample.

Figure 5a shows the estimated coating thickness profile in the reactor of batch B1 (350 °C), B2 (400 °C) and B3 (450 °C) from sample mass gain. The coating thickness estimated at 350 °C for sample S1 (250 nm) is close to the one observed in Fig. 2 by GD-OES (210 nm) and SEM (200 nm) in Fig. 4. The calculated thickness of the coating which contains less carbon, S1 at 400 °C (about 430 nm), is in good agreement with the one observed by SEM (about 430 nm) and quite in good agreement with the thickness estimated by GD-OES (about 500 nm). At 450 °C, the correlation between GD-OES (330 nm), SEM (320 nm) and mass measurement (300 nm) are in good agreement.

It is shown in Fig. 5 that the calculated coating thickness by mass difference increases with temperature from 350 to 400 °C then decreases at 450 °C. Moreover, the thickness homogeneity along the furnace length increases when the temperature decreases.

Figure 5b shows the evolution of the Co/Mn atomic ratio measured in the coating by GD-OES for S1 (25 cm) and S3 (50 cm) sample as a function of temperature. The $\:\frac{\left[Co\right]}{\left[Mn\right]}$ ratio is higher than the $\:\frac{\left[Co{\left(acac\right)}_{3}\right]}{\left[Mn{\left(acac\right)}_{3}\right]}$ ratio used in the solution for the deposition at 350 °C but is equal at 400 °C and 450 °C.

### Influence of pressure on the composition and thickness of Co-Mn oxide coatings

The XRD pattern of the coating formed at 350 °C at 4 and 10 mbar (Batch 9 and 10) (not shown here) shows that the pressure does not influence the nature of the coating: Mn_x_Co_1−x_O is formed at both pressures.

**Fig. 6:**
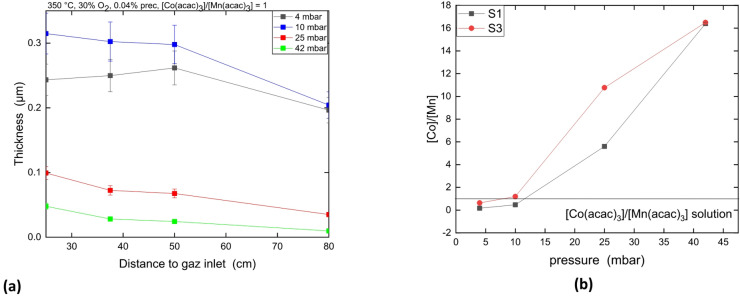
Coating thickness profile as a function of the distance to gas inlet at various pressure **(a)**. $\:\frac{\left[Co\right]}{\left[Mn\right]}$ atomic ratio measured in the coating by GD-OES as a function of pressure **(b)**.

Figure 6a presents the coating thickness profile estimated by mass difference along the tube furnace at different process pressure at 350 °C. The coating thickness increases with pressure from 4 to 10 mbar. At 25 mbar and above, the coating thickness drops drastically. At 350 °C, the deposition rate remains constant from 25 to 50 cm from the gas inlet for the entire pressure range used.

**Fig. 7:**
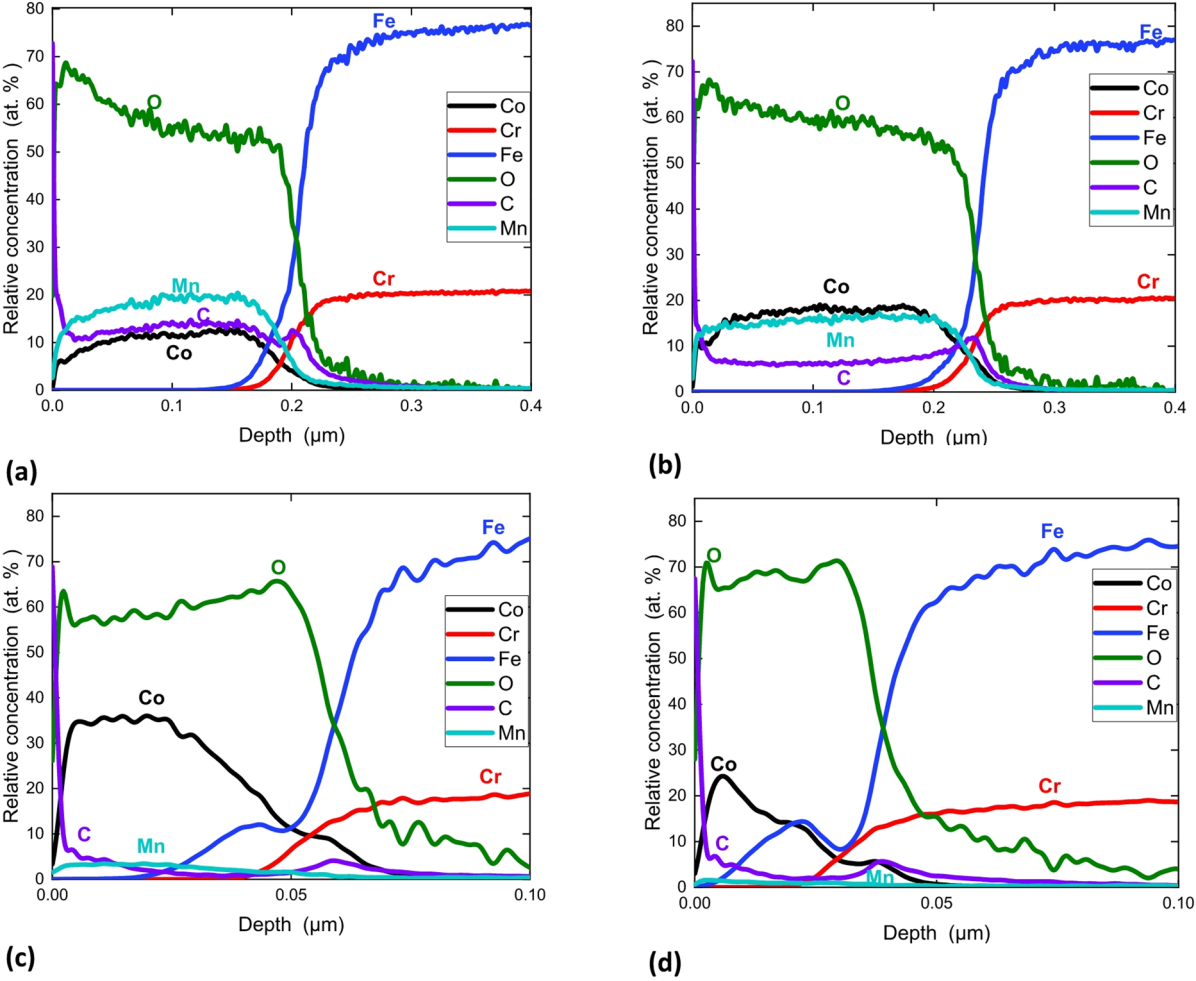
GD-OES quantitative depth profiles of the coating at 350 °C 30% O_2_, 0.04% precursor at a pressure of **(a)** 4 mbar **(b)** 10 mbar, **(c)** 25 mbar and **(d)** 42 mbar.

The $\:\frac{\left[Co\right]}{\left[Mn\right]}$ ratio in the coating is lower than the ratio of precursor in the solution at 4mbar, become equal at 10 mbar and increase with pressure (see Fig. 6b). Figure 7 presents the GD-OES analysis of every sample S3 from Batch 9, 10, 11 and 12. The GD-OES analysis of the coatings reveals that the carbon content in the coating decreases with the pressure increases from 4 mbar (15 at%), 10 mbar (6 at%). At 25 mbar and above, carbon proportion is about and 3 at%.

### Influence of O_2_ proportion on the composition and thickness of Co-Mn oxide coatings

**Fig. 8:**
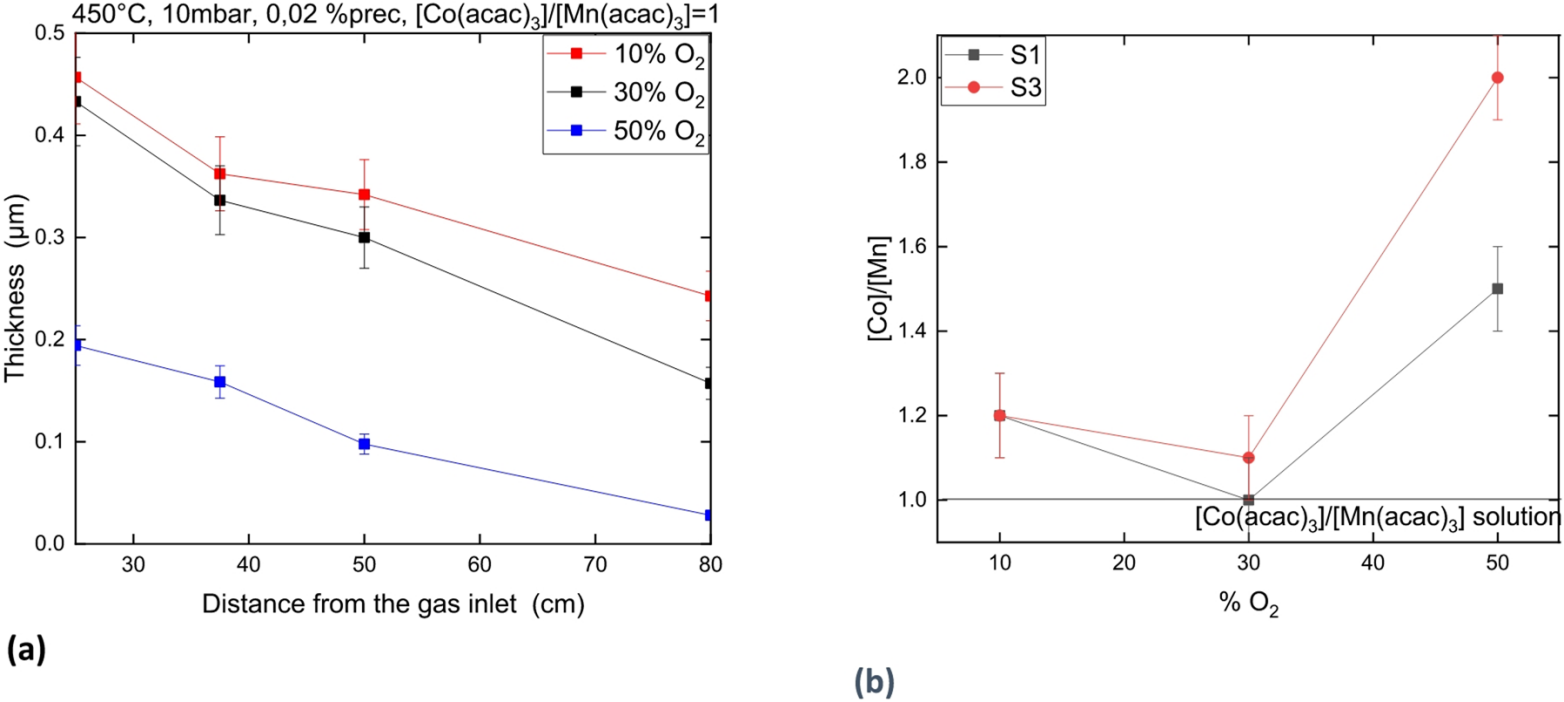
Coating thickness as a function of the distance to gas inlet (**a).** $\:\frac{\left[Co\right]}{\left[Mn\right]}$ ratiomeasured in the coating by GDOES as function of %O_2_ **(b).**

Figure 8a shows that the coating thickness at 450 °C decreases when the %O_2_ injected in the gas phase increases. Moreover, Fig. 8b shows that the $\:\frac{\left[Co\right]}{\left[Mn\right]}$ atomic ratio in the coating is quite constant from 10% to 30% of O_2_ in the gas phase but becomes significantly higher at 50% O_2_. In all cases, Mn_3−x_Co_x_O_4_ spinel structure with CoO cubic phase and low carbon concentration is formed.

### Influence of precursor proportion on the composition and thickness of Co-Mn oxide coatings

**Fig. 9:**
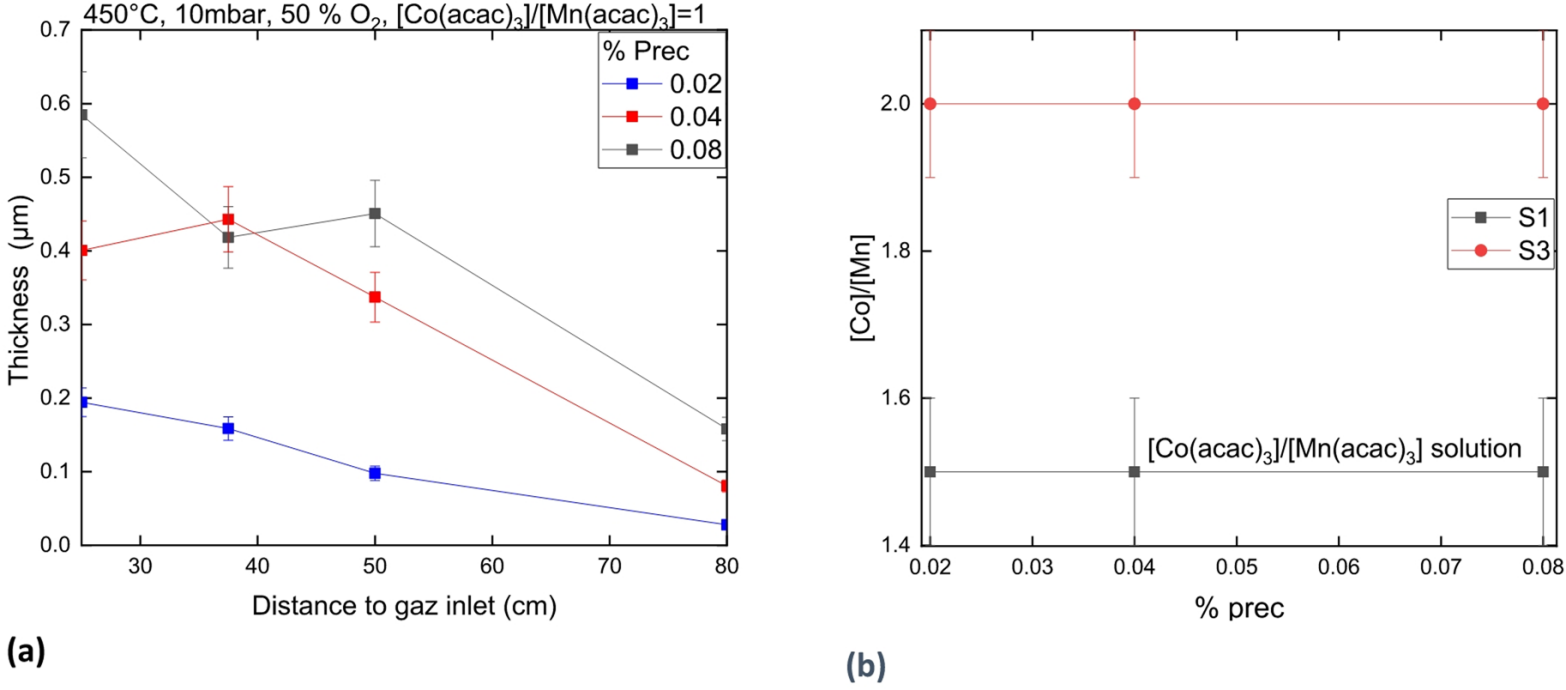
Coating thicknessas a function of the distance to gas inlet at various proportion of precursor in the gas phase **(a).** $\:\frac{\left[Co\right]}{\left[Mn\right]}$ atomic ratio measured in the coating by GDOES as function of the proportion of precursor in the gas phase **(b)**.

Figure 9a presents the profile thickness of the coating along the tube furnace regarding the precursor proportion in the gas phase at 450 °C. It is observed that the coating thickness increases when the precursor proportion in the gas phase increases from 0.02 to 0.04%, At 0.08% precursor in the gas phase, the coating thickness is hardly thicker than at 0.04%. While the chemical composition is not influenced (see Fig. 9b) the crystallographic structure shift from a Mn_2_CoO_4_ spinel + CoO cubic phases at 0.02 and 0.04% prec to a CoO cubic phase at 0.08% prec (XRD patterns are not shown here): all coatings have low carbon concentration (< 5 at%). The chemical composition with a higher proportion of Co compare to Mn is due to the high O_2_ concentration used in this test (50% of O_2_, see part b).

### Influence of $\:\frac{\left[Co{\left(acac\right)}_{3}\right]}{\left[Mn{\left(acac\right)}_{3}\right]}\:$ratio in the liquid solution on the composition and deposition rate of Co-Mn oxide coatings

**Fig. 10:**
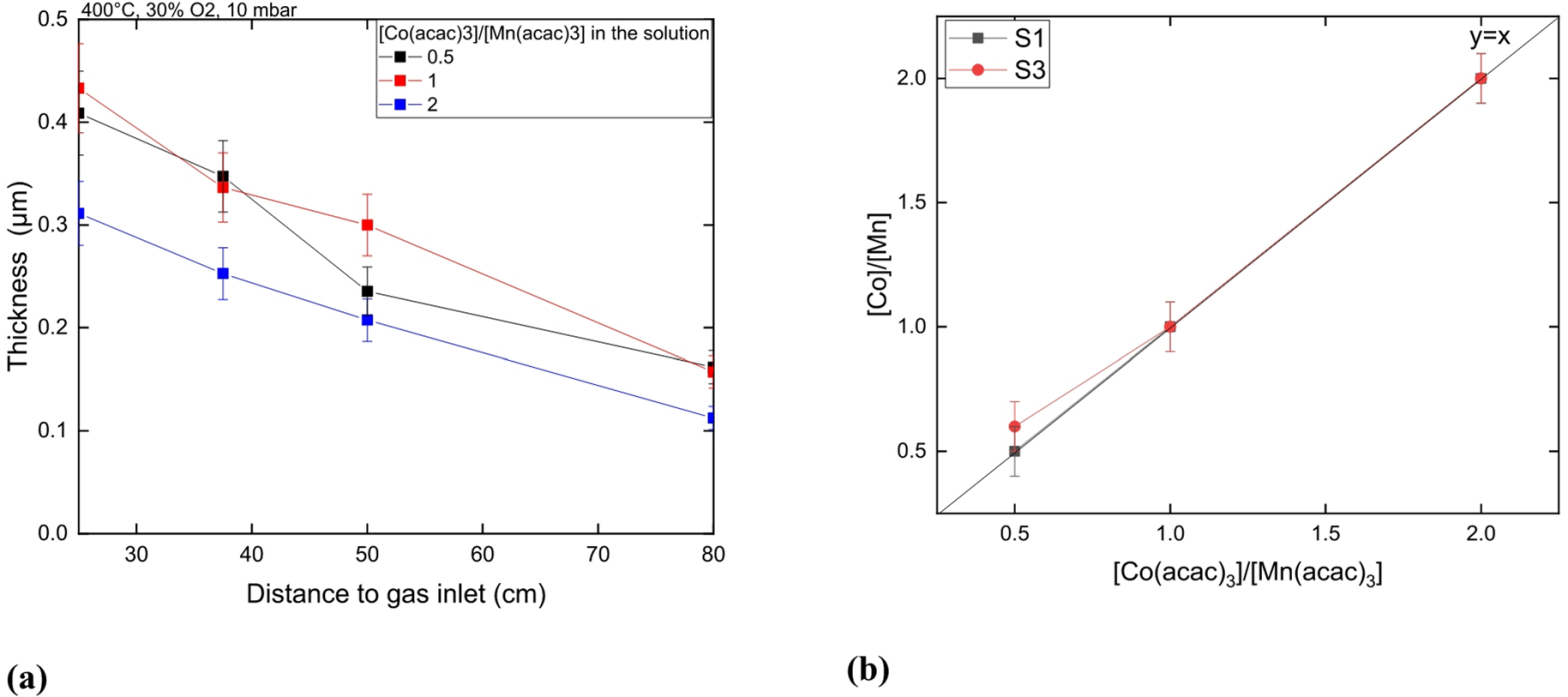
Coating thickness as function of the distance to gas inlet **(a)**, $\:\frac{\left[Co\right]}{\left[Mn\right]}$ atomic ratio measured in the coating by GDOES as a function of the proportion of precursor in the solution **(b)**.

Figure 10a presents the deposition rate profile along the tube furnace for different solution composition at 400 °C. All profiles are quite similar. The coating thickness is lower when the $\:\frac{\left[Co{\left(acac\right)}_{3}\right]}{\left[Mn{\left(acac\right)}_{3}\right]}$ ratio = 2, showing that the Co(acac)_3_ precursor is slowing down the deposition process. Figure 10b shows that in these experimental conditions (T, P, %O_2_), the $\:\frac{\left[Co\right]}{\left[Mn\right]}$ ratio in the coating is roughly equal to the $\:\frac{\left[Co{\left(acac\right)}_{3}\right]}{\left[Mn{\left(acac\right)}_{3}\right]}$ ratio in the solution. This composition remains from sample S1 to sample S3 so for at least 25 cm.

## Discussion

CVD process can be described as follows^15–17^:

- *Step 1*: *homogeneous reactions* (gas phase reaction) where the precursors react or thermally dissociate in the gas phase when temperature is high enough. These reactions make by-products, which may remain volatile. Nevertheless, the formation of powder and an impoverishment of the gas phase in precursor may happen if the reaction or thermal decomposition make nonvolatile by-products.
- *Step 2*: *deposition* where the precursors and the precursor by-products interact with the surface making physisorption. The deposition rate is theoretically directly proportional to the precursor partial pressure in the gas phase whatever the rate limiting process: by the reaction rate between the molecules and the surface (kinetic regime) or by the diffusion of the species from the gas phase to the surface (diffusion regime).
- *Step 3*: *reaction on the surface* where the adsorbed precursors or by-product precursors dissociate on the surface, react with each other or with reactant like O_2_ and form a solid compound or an oxide.

The rate of the phenomena is driven by Arrhenius law. So, the temperature has a major role in the gas phase and on the surface. In the gas phase, the homogeneous reactions rate also increase proportionally to the amount of precursor and to the amount of O_2_ in the gas phase. On the surface, the deposition rate also increases proportionally to the amount of precursor in the gas phase.

### Influence of T on the composition and deposition rate of the Co-Mn oxide coating

As observed previously (cf. 3.1), when the temperature increases from 350 to 400 °C, the growing rate increases according to a kinetic law driven by an Arrhenius law. But at higher temperatures, between 400 and 450 °C, the growing rate decreases. To explain this lower growth rate, it is proposed that the formation rate of powder, owing to homogeneous reaction, is faster at higher temperature, leading to a precursor impoverishment in the gas phase. This happens during the transport between the VapBox and the isotherm area. The increase of deposition rate between 400 and 450 °C does not appear to compensate this phenomenon. The faster powder formation is consistent with the lower deposition thickness homogeneity observed when the temperature increases.

The lower carbon concentration in the coating and the transformation from (Mn, Co)O to spinel when T is higher than 350 °C is explained by a faster reaction rate between O_2_ and the organo-metallic compounds at the surface of the sample at higher temperature. This allows a good C removal and a complete film oxidation while the film is growing.

### Influence of pressure on the composition and deposition rate of the Co-Mn oxide coating

The increase of total pressure has two contradictory effects on the coating growth rate according to the previous description of CVD processes. First, the increase of pressure lead to an increase of the deposition rate by increasing the amount of precursor in the gas phase. Nevertheless, it leads to gas phase impoverishment by enhancing the formation of powder as it is depicted in^18^. Powder formation removes precursors from the gas phase and, in consequence, decreases the deposition rate. This effect is observed in Fig. 6a: the deposition rate increases as long as the pressure is below 10 mbar but at 25 mbar and more, a drastic drop of growth rate is observed.

The increase of [Co] proportion in the coating at high pressure indicate that the impoverishment of precursor in the gas phase is more important for Mn(acac)_3_ than for Co(acac)_3_ leading to a gas phase with more Co(acac)_3_ than Mn(acac)_3_. So, the coating becomes richer in Co while the pressure increases.

The pressure does not impact the crystallinity of the coating but the C amount. The C concentration in the coating decreases with the pressure. In matter of fact, the increase in O_2_ partial pressure combined with a lower growth rate drive to a better C removal in the coating. Then, more O_2_ atoms are available to remove the carbon by-products. At higher pressure, the deposition rate decreases drastically letting even more O_2_ atoms to remove the C by-products and diminishing its encapsulation into the film. The same effect is observed in the sample S3 in comparison to sample S1 for all the batch relatives to pressure.

### Influence of O_2_ proportion on the composition and deposition rate of the Co-Mn oxide coating

Increasing the O_2_ partial pressure increases the homogeneous reaction between O_2_ and the precursor in the gas phase. In consequence, the formation of powder is enhanced which results in the impoverishment of the gas phase in precursor and to a decrease of the deposition rate (see Fig. 8). Interestingly, the formation of spinel occurs at T > 350 °C even at 10% O_2_, showing that the O_2_% is not the main parameter to make spinel (Mn_x_Co_3−x_O_4_) but the temperature.

At 50% O_2_, the film is enriched with Co losing the good agreement between the solution composition and the coating composition. Moreover, the homogeneity in composition between S1 and S3 is lost. Both observations suggest that increasing the O_2_ proportion drives the system to consume the Mn(acac)_3_ preferentially in the gas phase, which, in consequence, enriches the gas phase with Co(acac)_3_. Consequently, the Co transfer from the gas phase to the surface become more important than the Mn transfer.

### Influence of precursor proportion on the composition and deposition rate of the Co-Mn oxide coating

The increase in %prec in the gas phase leads to an increase in precursor concentration. From %prec = 0.02% to 0.04%, this parameter drives to a linear increase of the coating thickness. However, it is observed that the growth rate does not increase linearly above 0.04% prec in the gas phase. It is hardly faster at 0.08% than at 0.04% prec. It is proposed that the formation of powder in the gas phase is enhanced when the %prec is over 0.04%. Therefore, the excess of precursor consumed in the tube between the VapBox and the isotherm area is much faster at 0.08% prec than at 0.04% prec in the gas phase. The resulting amount of precursor in the gas phase when the gas front reaches the isotherm area seems to be very close at 0.08% and 0.04% prec in the gas phase.

## Conclusions

In the present study, the deposition of homogeneous and continuous Mn-Co oxide coating on ferritic stainless steel by DLI-MOCVD from a mixed solution of Co and Mn organometallic precursor is demonstrated in a portion of 25 cm inside a 1 m long hot wall tubular reactor. The main results are as follows:

- At 350 °C, cubic Mn_x_Co_1-x_O_y_ oxide is formed with high C content but at higher temperatures, typically 400 and 450 °C, spinel Mn_3-x_Cr_x_O_4_ with CoO is formed with a low C proportion < 5 at%.
- The $\:\frac{\left[Co\right]}{\left[Mn\right]}$ atomic ratio in the coating, the maximum deposition rate and its homogeneity along the tube furnace length depends on temperature, pressure and O_2_ in the gas phase.
- The best process conditions for making Mn-Co spinel coating on stainless steel interconnect for SOC application are: 400 to 450 °C, 10 to 30%O_2_, 4 to 10 mbar and 0.04%prec. These conditions allow the maximum thickness with the best homogeneity in composition and thickness along the tube furnace, a $\:\frac{\left[Co\right]}{\left[Mn\right]}$ ratio in the coating similar to that used in the solution and a minimum carbon concentration in the coating.

The performance of such coating (electrical properties and barrier against Cr volatilization) must be evaluated in SOC conditions.

## Supplementary Information

Below is the link to the electronic supplementary material.

Supplementary Material 1

## Data Availability

Data is provided within the manuscript or supplementary information files. The GD-OES are not added to the supplementary information files but can be query to the author by the reviewers is necessary. Please ask the Corresponding author if you need these data.
