# Supplementary material for: Mixed Co-Mn oxide coatings synthetized by DLI-MOCVD for SOC interconnect a parametric study for composition and homogeneity control
Source: Sci Rep. 2025 Nov 14;15:39953. doi: 10.1038/s41598-025-23783-5 (PMC12618656; doi:10.1038/s41598-025-23783-5)
Supplement: Supplementary file 1 — Supplementary Material 1 [file 41598_2025_23783_MOESM1_ESM.pdf]

Supplementary material as the data not shown in the text and subject to interpretation.

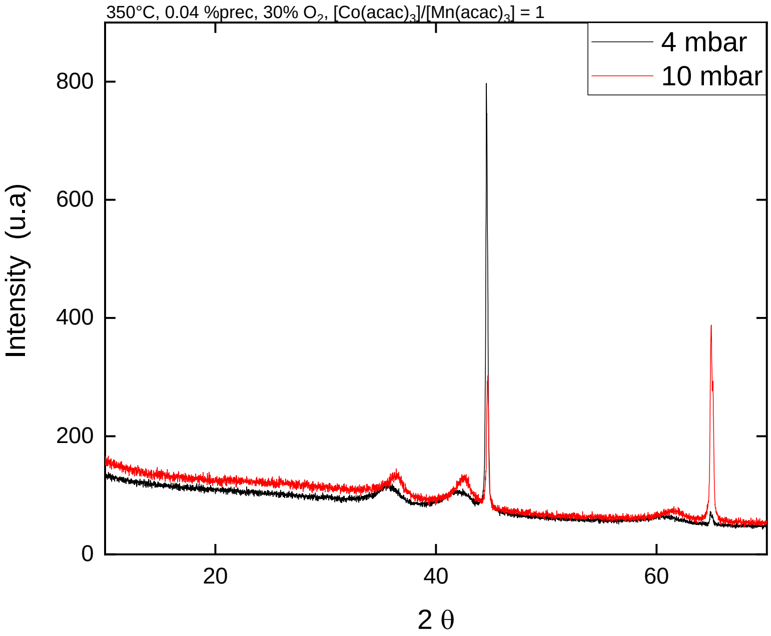

Add 1: XRD pattern of sample S3 of batch B9 and B10 showing that the difference of pressure does not influence a lot the crystallinity of the coating. The XRD pattern of batch B11 and B12 are very noisy owing to a combination of the substrate roughness and a too thin coating. So, nothing interesting could be extracted. The peaks at 45 and 65° are linked to the substrate. So, the difference of intensity is not discussed.

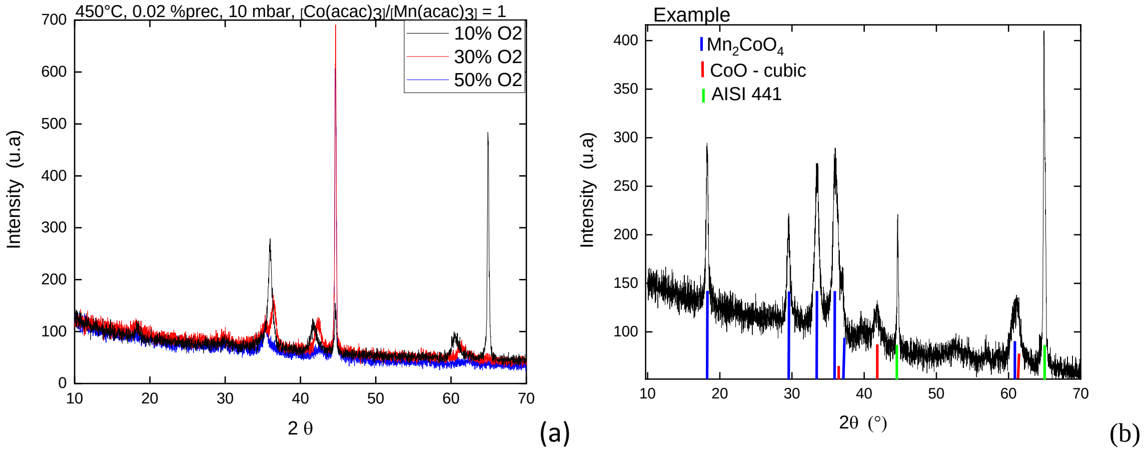

Add 2: **(a)** XRD pattern of S3 sample of batch B3, B6 and B13 showing that the XRD pattern at the different O_2 %. The peaks at 18 and 30 ° are very weak and the peaks around 35° are intertwined. We interpreted that signal as a mixture of Mn_2CoO_4 + CoO because the peak at 18° showing the presence of a spinel still exist despite the signal is weaker than in the example (b) presented in the manuscript.

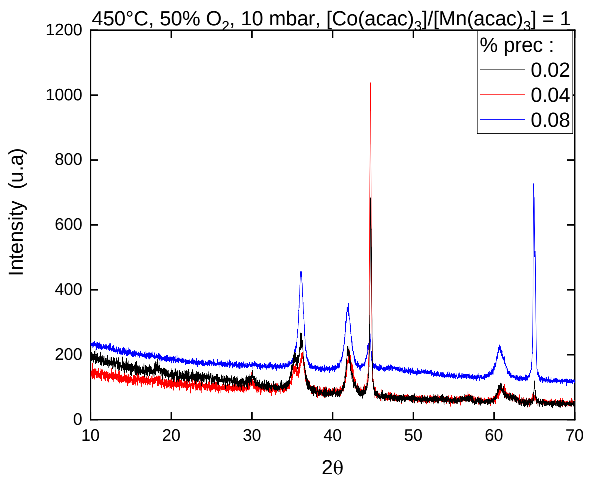

Add 3: XRD pattern of S3 sample of batch B4, B5 and B6 showing that the the XRD pattern is a Mn_2CoO_4 + CoO cubic and shift to a CoO cubic structure when the %prec is at 0.08%.

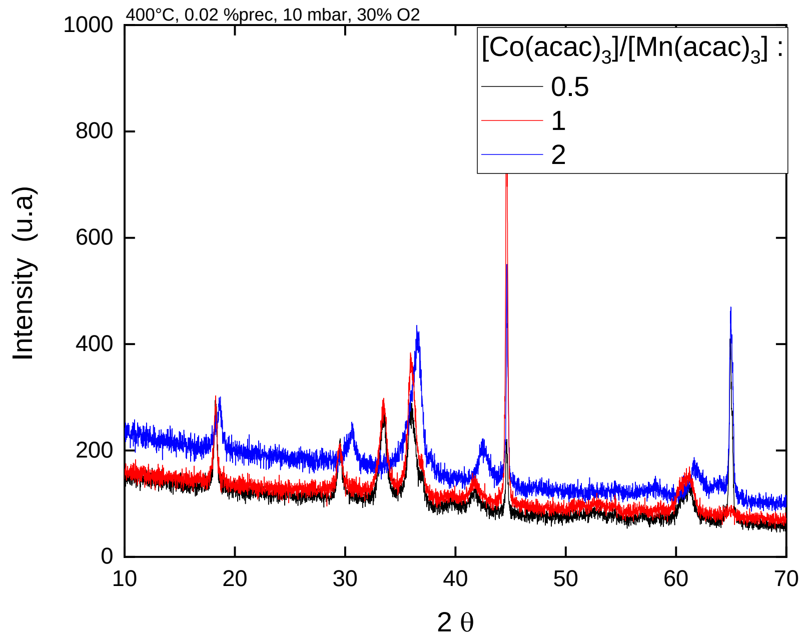

Add 4 : XRD pattern of S1 sample of batch B2, B7 and B8 showing that the the XRD pattern is a Mn_2CoO_4 + CoO cubic and shift to a MnCo2O4 + CoO cubic when the coating become Co rich.

Conversely to the XRD patterns which may be interpreted and discuss with the reviewers, the GD-OES not presented in the manuscript are very similar to the GD-OES presented. We choose to not share it except if the reviewers are very interested about these data.
